# Enzyme Activities of Five White-Rot Fungi in the Presence of Nanocellulose

**DOI:** 10.3390/jof7030222

**Published:** 2021-03-18

**Authors:** Carolina Reyes, Alexandre Poulin, Gustav Nyström, Francis W. M. R. Schwarze, Javier Ribera

**Affiliations:** 1 Laboratory for Cellulose & Wood Materials, Empa, Überlandstrasse 129, 8600 Dübendorf, Switzerland; alexandre.poulin@gmail.com (A.P.); gustav.nystroem@empa.ch (G.N.); 2 Department of Health Science and Technology, ETH Zürich, Schmelzbergstrasse 9, 8092 Zürich, Switzerland; 3 Laboratory for Cellulose & Wood Materials, Empa, Lerchenfeldstrasse 5, 9014 St. Gallen, Switzerland; Francis.Schwarze@empa.ch

**Keywords:** lignocellulosic enzymes, TEMPO, cellulose, CNC, CNF, *Trametes*, *Ganoderma*, *Rigidoporus*

## Abstract

White-rot fungi can degrade all lignocellulose components due to their potent lignin and cellulose-degrading enzymes. In this study, five white-rot fungi, *Trametes versicolor*, *Trametes pubescens*, *Ganoderma adspersum*, *Ganoderma lipsiense,* and *Rigidoporus vitreus* were tested for endoglucanase, laccase, urease, and glucose-6-phosphate (G6P) production when grown with malt extract and nanocellulose in the form of TEMPO (2,2,6,6-tetramethylpiperidine-1-oxyl radical) oxidized cellulose nanofibrils (CNF) and cellulose nanocrystals (CNC). Results show that temperature plays a key role in controlling the growth of all five fungi when cultured with malt extract alone. Endoglucanase activities were highest in cultures of *G. adspersum* and *G. lipsiense* and laccase activities were highest in cultures of *T. versicolor* and *R. vitreus*. Urease activities were highest in cultures of *G. adspersum*, *G. lipsiense,* and *R. vitreus*. Glucose-6-phosphate levels also indicate that cells were actively metabolizing glucose present in the cultures. These results show that TEMPO-oxidized CNF and CNC do not inhibit the production of specific lignocellulose enzymes by these white-rot fungi. The apparent lack of enzymatic inhibition makes TEMPO-oxidized CNF and CNC excellent candidates for future biotechnological applications in combination with the white-rot fungi studied here.

## 1. Introduction

White-rot fungi, with the ability to degrade lignocellulosic material, are valued in the paper, textile, and food industries for their ability to produce various lignocellulosic-degrading enzymes [1,2,3]. These fungi differ from brown-rot in that they can degrade all lignocellulose constituents (lignin, cellulose, and hemicellulose) producing CO_2_ as a byproduct of this metabolism [4,5]. Among the hydrolytic enzymes produced by white-rot fungi, to degrade the cellulose component of lignocellulosic material, are the endoglucanases. These enzymes are capable of cleaving the β-1,4-glycosidic linkages of glucose units that make up cellulose into cellobiose and cellulooligosaccharides [6]. Other hydrolytic enzymes used by white-rot fungi include: cellobiohydrolases, β-glucosidases, and lytic polysaccharide monooxygenases [6,7]. To degrade lignin, white-rot fungi use lignin peroxidase, manganese peroxidase, and laccase [8]. Laccases are a copper containing enzyme used by white-rot fungi to oxidize a variety of aromatic hydrogen polyphenols and non-phenolic compounds [8].

Previous studies have shown that the production of lignocellulosic enzymes by white-rot fungi depends on many factors like the strain of fungi, carbon and nitrogen substrate, and crystallinity of the cellulose [7,9,10,11,12,13]. In certain white-rot fungi, glucose and other simple sugar molecules can inhibit cellulase production [14,15]. Additionally, certain nitrogen compounds can stimulate cellulase and laccase production in some white-rot fungi [11,16,17]. Satyamurthy et al. [9] hypothesized that due to their highly anionic charge, cellulose nanocrystals prepared by sulfuric acid hydrolysis would inhibit fungal growth. In their study, the growth of *Trichoderma reesei* was inhibited by cellulose nanocrystals compared to when they were grown with cellulose prepared by microbial hydrolysis. In addition, the fungus produced less cellulases when grown with cellulose nanofibrils, prepared by high-pressure homogenization processes. However, other studies focused on the use of cellulose nanocrystals and nanofibrillated cellulose materials for bioengineering applications, have found these types of materials do not inhibit the metabolic activities of other types of microorganisms including cyanobacteria and algae [18].

In this study, our aim was to determine the effect of TEMPO (2,2,6,6-tetramethylpiperidine-1-oxyl radical) oxidized cellulose nanofibrils (CNF) and cellulose nanocrystals (CNC) on the endoglucanase, laccase and urease activities of five different white-rot basidiomycetes fungi when incubated with this material in malt extract medium. Additionally, glucose catabolism was investigated by analyzing glucose-6-phosphate (G6P) concentrations. TEMPO-oxidized CNF and CNC material were selected for this study because of their potential application in manufacturing bio-based hydrogels with wood decay fungi. As such, it would be important to determine whether the material is harmful to various filamentous fungi being considered for this future application. Additionally, it would be important to determine whether the fungi can produce enzymes that could potentially degrade TEMPO-oxidized CNF and CNC as an attractive bio-degradative feature. Given recent findings showing that the manufacturing cost of TEMPO-oxidized CNF can be reduced by decreasing TEMPO in the production process [19], the use of this material along with CNC for future biotechnological applications appears economically feasible. We hypothesized that TEMPO-oxidized CNF and CNC would not inhibit enzyme production by white-rot fungi. To our knowledge, this is the first study to characterize various white-rot basidiomycetes in the presence of TEMPO-oxidized CNF and CNC.

## 2. Materials and Methods

### 2.1. Fungal Growth Rate Experiments

Prior to initiating the growth experiments of fungi in the presence of cellulose, the optimal growth parameters were first established. Five fungal strains were used for all subsequent experiments mentioned in the manuscript including *Trametes versicolor* (Empa strain 159), *T. pubescens* (Empa strain 220), *Ganoderma adspersum* (Empa strain 003), *G. lipsiense* (Empa strain 646) [20,21], and *Rigidoporus vitreus* (Empa strain 643) [22,23]. The taxonomic identity of the fungi is based on mycobank (http://www.mycobank.org, accessed on 15 February 2021), the most frequently used online registry for nomenclature repositories. Fungi were grown in malt extract agar [MEA] (Oxoid) or glucose agar at 1%, 2%, and 4% (*w/v*). They were incubated at various relative humidity levels (60%, 70%, and 80%) and temperatures (15 °C, 20 °C, 25 °C, and 30 °C). The pH experiments were conducted in 2% (*w/v*) MEA at 80% relative humidity and 25 °C. The pH of the media was adjusted with 1 M HCl and 0.1 M NaOH. The media was prepared using sterile, distilled water, and all fungal cultures were incubated in the dark.

### 2.2. Fungal Enzyme Experiments

For the enzymatic experiments, fungi were grown in 70 mL liquid malt extract media (ME) supplemented with 1 g TEMPO-oxidized CNF (1.2 wt%) and sulfuric acid hydrolyzed CNC (15 wt%) giving a final CNF and CNC concentration of 0.017 wt% and 0.2 wt% respectively. The TEMPO-oxidized CNF was prepared as previously described [24,25] and added to commercially purchased cellulose nanocrystals (CNC) (Celluforce, Canada). The media was prepared using water with a resistivity ≥ 18 MΩ cm^−2^. Cultures that were shaken throughout the experiment were continuously agitated at 120 rpm in the dark. Cultures left standing were initially agitated at 120 rpm for 2 days, to promote the growth of the fungi, and then left to stand in the dark for the rest of the experiment. At different time intervals, 1.5 mL of supernatant was removed, centrifuged at 10,000 rpm for 10 min to remove any cell biomass. The centrifuged supernatants were transferred to new test tubes and the tubes were frozen at −20 °C for further analysis.

### 2.3. Endoglucanase Assay

The following method is based on modifications to previously described methods [26,27,28]. Fifty microliters of sample (undiluted or diluted in 50 mM acetate buffer pH 5) were mixed with 450 μL of 0.05% (*w*/*v*) medium viscosity carboxymethylcellulose (CMC) reagent (Sigma-Aldrich, Buchs, Switzerland) and the tube heated to 50 °C for 10 min. A substrate blank was also prepared consisting of 450 μL CMC and 50 μL acetate buffer. Afterward, 100 μL of this mixture was transferred to a new tube and to this, 200 μL of 1% (*w*/*v*) cyanoacetamide and 1 mL of borate (100 mM pH 9) was added. The samples were heated to 80 °C and then left to cool for at least 40 min before proceeding to measure the ultraviolet (UV) absorbance at the wavelength of 276 nm using a quartz cuvette and a Genyses 10S UV visible spectrophotometer. Glucose standards (20 mM stock solution) were prepared in 50 mM acetate buffer (pH 9) including: 0 mM, 0.1 mM, 0.25 mM, 0.5, and 1–6 mM standards. Following preparation of the standards, 100 μL of the standard solution was transferred to a new tube, and 1% (*w*/*v*) cyanoacetamide and borate were added as described above for the samples. Enzyme activities are reported as international units (IU) which are defined as the amount of enzyme that releases 1 μmol of reducing sugars per min. The IU was determined by converting mM of reduced glucose to μmol/mL and the resulting value was divided by the assay time.

### 2.4. Laccase Assay

This method is based on previously published methods [29,30]. For this assay, 700 μL of citrate buffer (pH 4.5) and 100 μL of 2,2′-azino-bis(3-ethylbenzothiazoline-6-sulfonic acid) [ABTS] were added to a quartz cuvette and then 200 μL of sample (undiluted or diluted in citrate buffer) was added to the cuvette. The mixture was pipetted up and down and the UV absorbance was immediately measured using a Genyses 10S UV visible spectrophotometer. Samples were measured at 10 s intervals for 3 min at the wavelength of 420 nm. Enzyme activities are reported as units per liter (U/L) and were calculated using the following formula:

$$\frac{U}{L}=\;\frac{\left(\Delta E\cdot Vt\right)}{\epsilon \;x\;d\;\cdot \;Vs}$$

where Δ*E* is the change in the extinction of light [min^−1^] at the wavelength of 420 nm, *ɛ* is the molar absorption coefficient of ABTS at pH 9 [36 mM cm^−1^], d is the thickness of the quartz cuvette (1 cm), *Vt* is the total volume measured, and vs. is the volume of the enzyme stock solution.

### 2.5. Urease Assay

Urease activities in standing cultures were investigated using the Urease Activity Assay Kit (Sigma-Aldrich, Buchs, Switzerland). Briefly, the kit quantitates ammonia produced from the hydrolysis of urea by urease present in samples. Ammonia, in turn, is revealed by the Berthelot method [31] by measuring the absorbance of the sample at the wavelength of 670 nm. One unit of urease corresponds to the amount of enzyme that catalyzes the formation of 1 μmole of ammonia per min at pH 7.

### 2.6. Glucose-6-Phosphate Assay

Glucose catabolism was studied in standing cultures by measuring G6P, one key intermediate for glucose transport into cells, using the Amplite™ Colorimetric Glucose-6-Phosphate Assay Kit (AAT Bioquest^®®^, LubioScience GmbH, Zürich, Switzerland) according to the manufacturer’s instructions.

## 3. Results

### 3.1. Growth Optima T. versicolor 159

**Figure 1:**
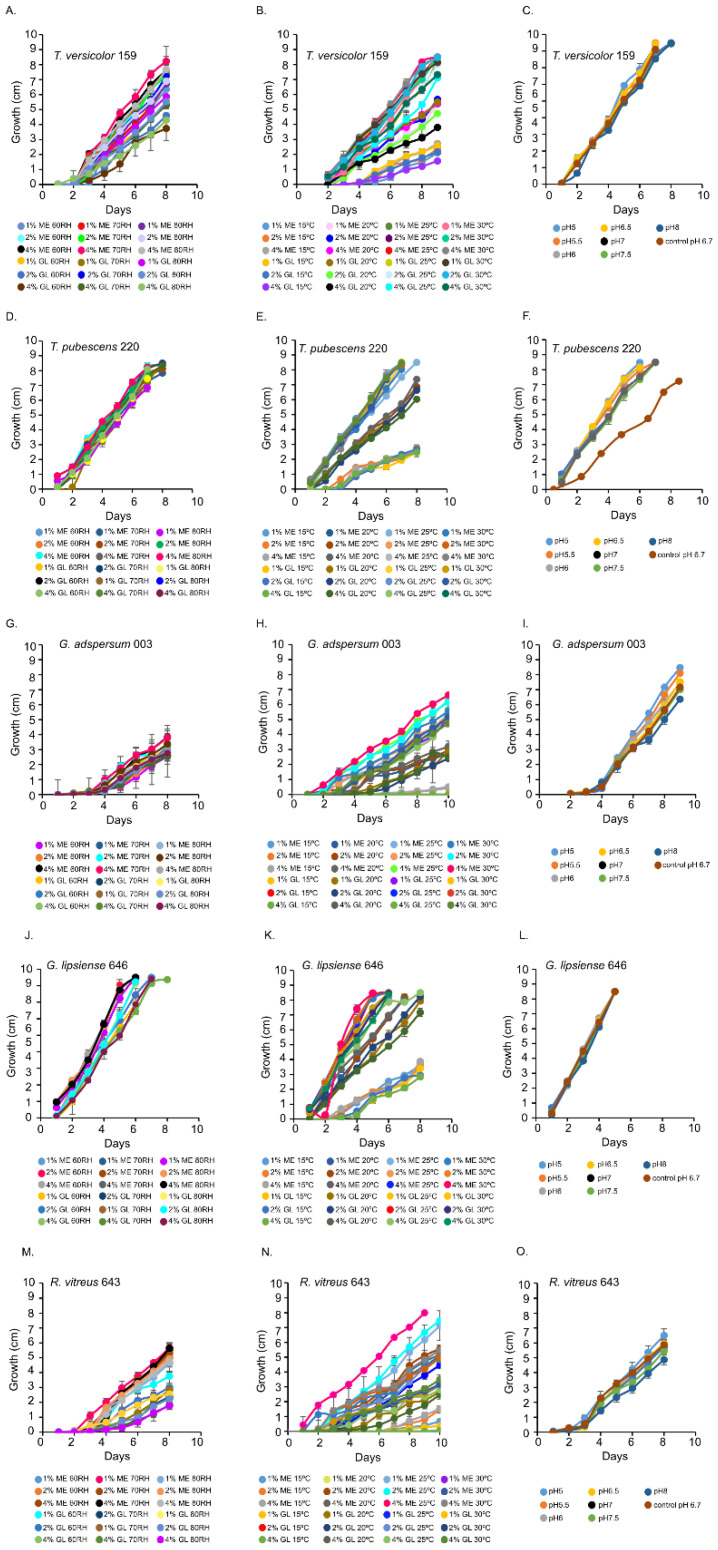
Growth rates of white rot fungi in the presence of different percentages of malt extract (ME) and glucose (GL). (**A**,**D**,**G**,**J**,**M**) show the growth of fungi at various percent relative humidities (RH). (**B**,**E**,**H**,**K**,**N**) show the growth of fungi at various temperatures. (**C**,**F**,**I**,**L**,**O**) show the growth of fungi at various pHs. Data represent an average of ≥ 5 biological replicates.

*T. versicolor* 159 grew optimally at 70% and 80% relative humidity in 1 and 2% (*w*/*v*) MEA (Figure 1A and Figure S1A–C). In the temperature experiments, its growth rate was highest at 25 °C and 30 °C (Figure 1B and Figure S1D–G). The pH curves for *T. versicolor* 159 were similar indicating that this factor is not critical for growth in 2% (*w*/*v*) MEA at 80% relative humidity and 25 °C (Figure 1C and Figure S1H).

### 3.2. Growth Optima T. pubescens 220

*T. pubescens* 220 grew well at all relative humidity levels in MEA or glucose (Figure 1D and Figure S2A–C). In the temperature experiments, *T. pubescens* 220 grew best at 25 °C and 30 °C in 2 and 4% (*w*/*v*) MEA and in 1 and 2% (*w*/*v*) glucose (Figure 1E and Figure S2D–G). With the exception of pH 6.7, *T. pubescens* 220 grew optimally at all pHs (Figure 1F and Figure S2H).

### 3.3. Growth Optima G. adspersum 003 and G. lipsiense 646

*G. adspersum* grew optimally at all relative humidities in MEA and glucose (Figure 1B and Figure S3A–C). In the temperature experiments, its growth rate was highest at 25 °C or 30 °C in 2% and 4% (*w/v*) MEA (Figure 1F and Figure S3D–G). *G. lipsiense* 646 grew best at 60% and 80% relative humidity in 4% (*w/v*) MEA and in 1% and 2% (*w/v*) glucose (Figure 1J and Figure S4A–C). The growth rate of *G. lipsiense* 646 was highest at 30 °C also when grown in 4% (*w/v*) MEA and 1 and 2% (*w/v*) glucose (Figure 1K and Figure S4D–G). Both *Ganoderma* spp. grew optimally at all pHs (Figure 1I,L; Figures S3H and S4H).

### 3.4. Growth Optima R. vitreus

*R. vitreus* 643 had the highest growth rate at 70% relative humidity (Figure 1M and Figure S5A–C). In the temperature experiments, it grew best at 25 °C in 4% (*w/v*) MEA (Figure 1N and Figure S5D–G). It grew optimally at pH 5 (Figure 1O and Figure S5H).

### 3.5. Endoglucanase Assays

**Figure 2:**
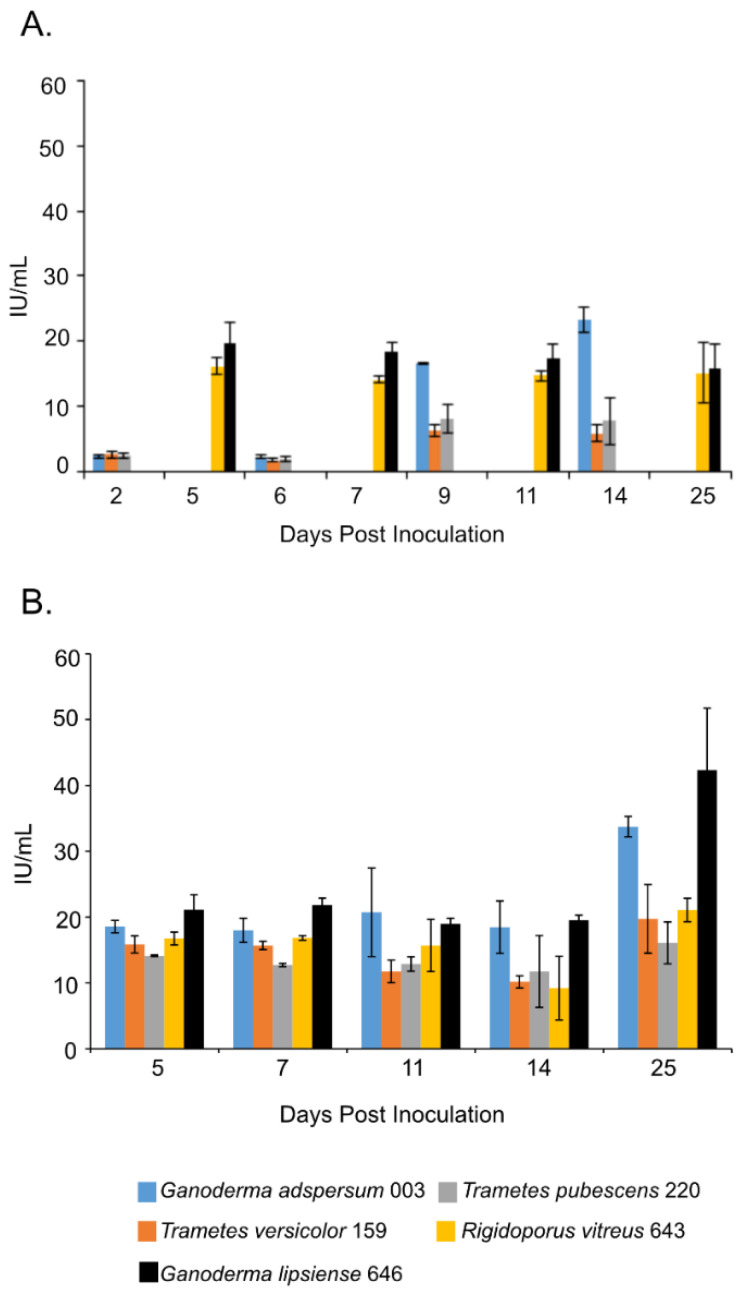
Endoglucanase activity at different days of incubation period of various white rot fungi when incubated with 2% (*w/v*) malt extract and TEMPO-oxidized CNF(0.017 wt%) and CNC(0.2 wt%) at 25 °C and 80% relative humidity. (**A**) Cultures were shaken at 120 rpm during incubation. (**B**) Cultures were initially shaken to stimulate growth but after 2 days they were left to stand for the duration of the experiment. Data represent an average of three biological replicates.

**Figure 3:**
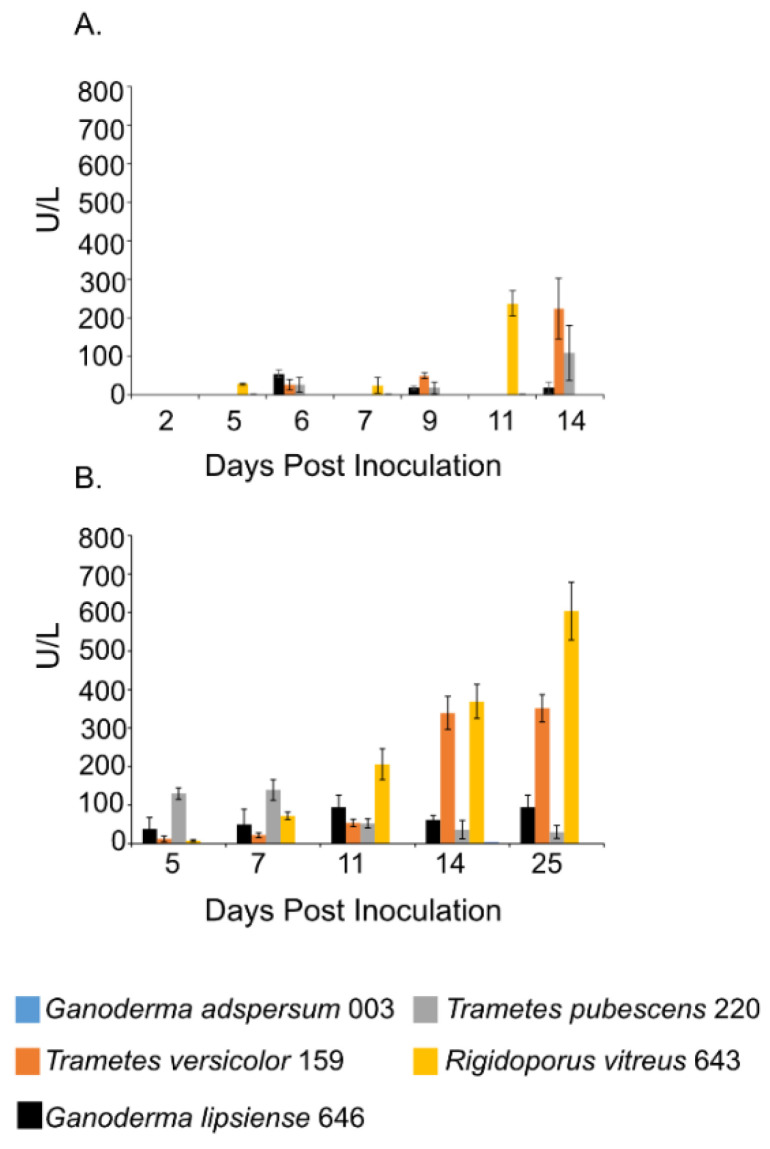
Laccase activity at different days of incubation period of various white rot fungi when incubated with 2% (*w/v*) malt extract and TEMPO-oxidized CNF(0.017 wt%) and CNC(0.2 wt%) at 25 °C and 80% relative humidity. (**A**) Cultures were shaken at 120 rpm during incubation. (**B**) Cultures were initially shaken to stimulate growth but after 2 days they were left to stand for the duration of the experiment. Data represent an average of three biological replicates.

Overall, samples that were shaking throughout most of the experiment in the presence of TEMPO-oxidized CNF (0.017 wt%) and CNC (0.2 wt%) had the lowest endoglucanase activities compared to those that were cultured under non-shaking conditions (Figure 2A,B). For example, at the end of the experiment, shaking cultures of *G. adspersum* 003, *G. lipsiense* 646, and *R. vitreus* 643 showed the highest endoglucanase activity (~23 ± 2 U/mL, ~16 ± 4 U/mL and ~15 ± 5 U/mL respectively) compared to *T. versicolor* 159 (~6 ± 2 U/mL) and *T. pubescens* 220 (~ 8 ± 4 U/mL). Under non-shaking conditions, *G. lipsiense* 646, *G. adspersum* 003, and *R. vitreus* 643 had the highest endoglucanase activity (Figure 3B). At the end of the experiment, the enzyme activities of *G. lipsiense* 646, *G. adspersum* 003, and *R. vitreus* 646 were ~42 ± 9 U/mL, ~34 ± 2 U/mL, and ~21 ± 1 U/mL respectively. Enzyme activities of. *T. versicolor* 159 (~20 ± 5 U/mL) and *T. pubescens* 220 (16 ± 3 U/L) were lower in comparison to the above fungi.

### 3.6. Laccase Assays

Laccase activity assays showed a similar trend as the endoglucanase experiments. Samples that were shaken had lower laccase activities compared to those that were left standing throughout the experiment (Figure 3A,B). *R. vitreus* 643 and *T. versicolor* 159 had the highest laccase activity under both conditions. Under shaking conditions at the end of the experiment, enzyme activities for *R. vitreus* 643 and *T. versicolor* 159 were ~238 ± 32 U/L and 225 ± 79 U/L. This is in contrast to the lower enzyme activities of *T. pubescens* 220 (~110 ± 71 U/L), *G. adspersum* 003 (~19 ± 14 U/L), and *G. lipsiense* 646 (~2 ±1 U/L). At the end of the experiments under static conditions, their activities were ~603 ± 75 U/L and ~351 ± 35 U/L respectively. In comparison, *G. adspersum* 003, *T. pubescens* 220, and *G. lipsiense* 646 had lower enzyme activities (~95 ± 31 U/L, ~30 ± 16 U/L, and ~4 ± 0 U/L respectively).

### 3.7. Urease Assay

**Figure 4:**
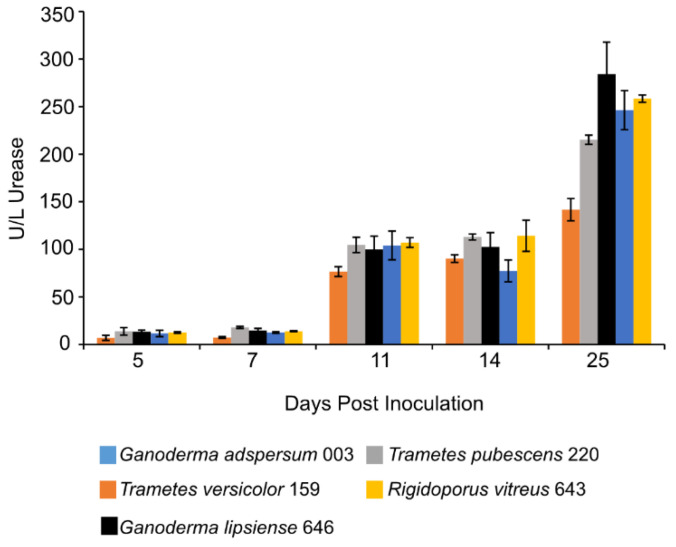
Urease activity at different days of incubation period of various white rot fungi when incubated with 2% (*w/v*) malt extract and TEMPO-oxidized CNF(0.017 wt%) and CNC(0.2 wt%) at 25 °C and 80% relative humidity. Cultures were initially shaken to stimulate growth but after 2 days they were left to stand for the duration of the experiment. Data represent an average of three biological replicates.

At 11 days post-inoculation, the activity of urease in all standing culture samples increased and remained stable through day 14 (Figure 4). After 25 days, urease activity was highest in cultures of *G. adspersum* 003 (284 ± 36 U/L), G. *lipsiense* 646 (246 ± 20 U/L), and *R. vitreus* 646 (258 ± 4 U/L).

### 3.8. Glucose Assay

**Figure 5:**
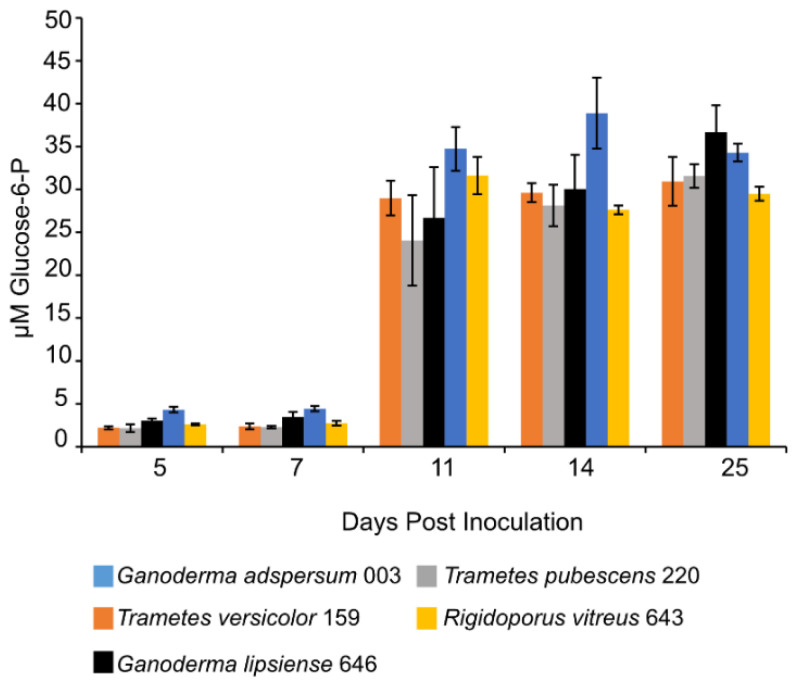
Glucose-6-P concentration at different days of incubation period of various white rot fungi when incubated with 2% (*w/v*) malt extract and TEMPO-oxidized CNF(0.017 wt%) and CNC(0.2 wt%) at 25 °C and 80% relative humidity. Cultures were initially shaken to stimulate growth but after 2 days they were left to stand for the duration of the experiment. Data represent an average of three biological replicates.

Concentrations of G6P increased in all standing cultures at 11 days post-inoculation and remained constant throughout the experiment (Figure 5). G6P concentrations ranged between 29 ± 1 μM measured in cultures of *R. vitreus* 643 and 36 ± 3 μM measured in cultures of *G. adspersum* 003.

## 4. Discussion

In this study, we addressed the hypothesis that TEMPO-oxidized CNF and CNC would not interfere with enzyme production by five white-rot fungi when exposed to this material during cell growth. To this end, we first optimized the growth conditions of the fungi on MEA before exposing fungi to TEMPO-oxidized CNF and CNC. Results from the growth experiments on MEA support findings from previous studies [32,33,34,35] showing that MEA, temperatures between 25–30 °C, and a broad pH range are optimal growth conditions for *T. versicolor* 159, *T. pubecens* 220, *G. adspersum* 003, *G. lipsiense* 646, and *R. vitreus* 643 strains. Temperatures ≤ 20 °C slowed down the growth of these fungi irrespective of the media demonstrating that temperature is a key factor controlling their growth and activity. Previous studies have shown that the type of carbon and nitrogen sources and their concentrations can influence the mycelial growth and polysaccharide production by white-rot fungi [36,37]. In addition to maltose, other carbohydrates typically present in MEA include fructose, glucose, and sucrose [38]. MEA is also rich in nitrogenous compounds like peptides, tryptophan, tyrosine, and vitamins [38,39]. Thus, the higher growth rates observed when fungi were grown in MEA vs. the glucose media were likely due to the greater availability of carbon and nitrogen substrates present in the MEA.

Having established the optimal growth conditions, we analyzed the activity of various enzymes in cultures incubated with 2% (*w/v*) MEA TEMPO-oxidized CNF (0.017 wt%) and CNC (0.2 wt%). Endoglucanase activity was analyzed due to its ability to cleave the β-1-4 linkages of cellulose [6]. The higher endoglucanase activities observed in the cultures left to stand compared to those that were left shaking throughout the duration of the experiments could be due to greater contact time between the fungal cells and cellulose. Fungal cells left to stand would have uninterrupted contact with the TEMPO-oxidized CNF and CNC material compared to shaking cultures. Better cell contact with the cellulose material could in turn trigger increased enzyme production associated with cellulose degradation. A similar hypothesis was put forward in studies involving lignin degradation by standing cultures of white-rot fungi [40]. Another explanation is that cells that are cultured under shaking conditions during growth are more dispersed and fragmented compared to cells that are left standing and this morphological difference could influence enzyme production. The influence of fungal morphology on the fungal secretion of products has been shown to vary between fungi [41]. Some fungi produce more of one product type when left undisturbed during growth while others produce more enzymes in the same conditions. Alternatively, shaking conditions could have lowered endoglucanase activity. Previous studies spanning several decades of research have observed that shaking or agitation can decrease cellulase activities in extracts from fungi [42,43,44] or in the culture producing the cellulase [45]. Various hypotheses have been put forward as to why this may occur, including possible shear stress on the cellulase.

Comparison of endoglucanase activities in the presence of TEMPO-oxidized CNF and CNC with other white-rot fungi exposed to cellulose materials showed the fungi in this study produced endoglucanase levels comparable to those in the Metreveli et al. [46] and Liu et al. [47] studies. The white-rot fungi in this study also produced higher levels of endoglucanase than those reported in the Satyamurthy et al. 2016 study (Table 1). Thus, TEMPO-oxidized CNF and CNC did not appear to inhibit endoglucanase production by white-rot fungi in this study. On the contrary, it appears to stimulate enzyme production comparable to lignocellulosic material.

Another enzyme that we analyzed for activity in cultures was laccase. Laccases are multi-copper oxidases that oxidize phenolic and non-phenolic substrates with the concomitant reduction of oxygen to water. Organic and inorganic substrates of this enzyme include ortho-, meta-, and para-substituted compounds with a lone electron pair [29]. Although extracellular laccases are constitutively produced in basidiomycetes under aerobic conditions [50], their production and activities are further stimulated by a variety of compounds including lignin or lignin derivatives such as ferulic acid, guaiacol, veratryl alcohol [51] and also by cellulose [13]. Similar to the endoglucanase results, laccase activities were highest in cultures that were standing compared to those that were shaken throughout the cultivation period. The highest activities of laccase were observed in standing cultures of *T. versicolor* 159 (351 U/L) and *R. vitreus* 643 (603 U/L) (Figure 3A,B). In the case of *T. versicolor* 159, this value is similar to activities obtained with *T. versicolor* (CBS100.29) cultured with grape seeds (250 U/L) or grape stalks (450 U/L) [52] and *T. versicolor* 145 cultured with mandarin peels (428 ± 19 U/L) (Table 2). Laccase production was higher than observed with the same *T. versicolor* 159 Empa strain in standing cultures grown with glucose and veratryl alcohol (122 ± 24 U/L) or spruce wood sawdust (198 ± 44 U/L) (Table 2). Additionally, the laccase activities of *R. vitreus* 643 in standing cultures (603 ± 75 U/L) were similar to those observed when the same culture was grown with spruce wood sawdust (755 ± 148 U/L) [53]. These results show that TEMPO-oxidized CNF and CNC stimulates laccase production in *T. versicolor* 159 and *R. vitreus* 643 similar to more complex lignocellulosic substrates. Various fungi possess different forms (isoforms) of laccases that are expressed depending on the substrate that these fungi encounter (Kaczmarek et al. [54]). Of the available white-rot fungal genomes, the genomes of *T. versicolor* and *T. pubescens* show that these fungi possess 24 and 8 laccase genes respectively (Table S1). It could be that the greater number of laccase genes in *T. versicolor* give it a competitive advantage over other white-rot fungi with less laccase genes if more of these isoforms are produced in response to lignocellulosic compounds. It would be interesting to determine in future experiments which genes, of the available fungal genomes, are expressed in the presence of TEMPO-oxidized CNF and CNC.

Previous studies have shown that depending on the concentration and type of nitrogen source (organic or inorganic) provided to white-rot fungi during growth, it can either antagonize or stimulate the production of lignocellulolytic enzymes [11,56,57,58] Ureases are nickel-containing enzymes that hydrolyze urea into ammonia and carbamate during nitrogen metabolism in fungi and other prokaryotes [59,60]. Increasing concentrations of urease in all standing cultures show that cells were making use of urea. Fungal cells were likely metabolizing nitrogen compounds present in the malt extract. Under the conditions in this study, both *Ganoderma* strains and *R. vitreus* appear to have benefited most from the presence of urea (Figure 5). These fungi could have been using the nitrogen source for either biomass growth or synthesis of lignocellulolytic enzymes.

Detection of low levels of G6P in culture supernatants (Figure 5) implies that cell lysis occurred to some extent during sampling or centrifugation steps. Moreover, these low concentrations of G6P suggest that glucose present in the media was metabolized by fungal cells. G6P is the product of phosphorylation of glucose to G6P by the enzyme hexokinase as part of the glycolysis pathway in prokaryotic cells [61]. In this study, the metabolized glucose could have come from the malt extract media [38] or it could have been produced from the breakdown of cellobiose to glucose by β-glucosidase enzymes outside the cell [6,62]. During white-rot cellulose degradation, cellulose is first degraded outside the fungal cell to cellulooligosaccharides (by endoglucanases). Next, cellulooligosaccharides are degraded into cellobiose (by cellobiohydrolases) and finally into glucose by β-glucosidases [63]. Glucose is then taken up into cells where it undergoes further metabolism via the glycolysis pathway for cell energy production in the form of ATP [61]. High glucose concentrations (e.g., >1 mM) have been shown to inhibit enzymes in white-rot fungi involved in cellulose degradation including β-glucosidases [62,64,65]. Our results show that the presence of glucose did not interfere with endoglucanase production by all five white-rot fungi (Figure 2).

These results highlight the potential use of these white-rot fungi in biotechnological applications involving TEMPO-oxidized CNF and CNC as a substrate. The white-rot fungi in this study could be used for biodegradation of TEMPO-oxidized and CNF materials, for example [66]. Another possibility is using these white-rot fungi to stimulate laccase and urease production and incorporation into TEMPO-oxidized materials for energy production [67,68].

## 5. Conclusions

The lack of inhibition of endoglucanase, laccase, and urease observed in this study when the white-rot fungi *T. versicolor* 159, *T. pubescens* 220, *G. adspersum* 003, *G. lipsiense* 646, and *R. vitreus* 643 were grown in the presence of malt extract and TEMPO-oxidized CNF and CNC, shows that TEMPO-oxidized cellulose materials are biocompatible with the fungi studied here. Endoglucanases are likely involved in the cleavage of β-1, 4-glycosidic linkages of CNF and CNC. Urease activities indicate active nitrogen uptake from the malt extract media. Glucose concentrations appear too low to inhibit cellulase activities in standing cultures. Cultivation under long-term static conditions resulted in higher enzyme production possibly due to greater contact time between cells and cellulose material. These results indicate that TEMPO-oxidized CNF and CNC would be an excellent candidate for bioremediation or enzymatic applications involving these fungi.

## Figures and Tables

**Table 1:** Comparison of endoglucanase activities of fungi grown in the presence of crystalline cellulose or wood.

Fungus	Growth Conditions	Endoglucanase Activity	Study
*Pycnoporus. coccineus* 310 (Basidiomycota)	1.5% micro crystalline cellulose (Avicel medium) (150 rpm, 27 °C)	65.6 ± 7.3 U/mL (14 days)	Metreveli et al. [46]
*Schizophyllum commune* 632 (Basidiomycota)	1.5% micro crystalline cellulose (Avicel medium) (150 rpm, 27 °C)	28 ± 3.9 U/mL (14 days)	Metreveli et al. [46]
*Trametes hirusta* 17 (Basidiomycota)	1.5% micro crystalline cellulose (Avicel medium) (150 rpm, 27 °C)	34.3 ± 3.1 U/mL (14 days)	Metreveli et al. [46]
*Irepex lacteus* 104 (Basidiomycota)	1.5% micro crystalline cellulose (Avicel medium) (150 rpm, 27 °C)	51.7 ± 4.3 U/mL (14 days)	Metreveli et al. [46]
*Trichoderma viride* (Ascomycota)	2% commercial microcrystalline cellulose (Mandel’s medium) (150 rpm, 30 °C)	~ 22 U/mL (15 days)	Liu et al. [47]
*Aspergillus niger* (Ascomycota)	2% commercial microcrystalline cellulose (Mandel’s medium) (150 rpm, 30 °C)	~ 9 U/mL (15 days)	Liu et al. [47]
*Trichoderma koningii* (Ascomycota)	2% commercial microcrystalline cellulose (Mandel’s medium) (150 rpm, 30 °C)	~ 32 U/mL (15 days)	Liu et al. [47]
*Trichoderma reseei* (Ascomycota)	2% commercial microcrystalline cellulose (Mandel’s medium) (150 rpm, 30 °C)	~5 U/mL (15 days)	Liu et al. [47]
*Trichoderma reseei* (Ascomycota)	Nanocellulose prepared by microbial hydrolysis (Mandel’s medium)	~0.04 IU/mL (Day 1)~0.16 IU/mL (Day 5)	Satyamurthy et al. [9]
*Trichoderma reseei* (Ascomycota)	Nanofibrilliated cellulose (NFC)	~0.02 IU/mL (Day 1)	Satyamurthy et al. [9]
*Aspergillus niger* (Ascomycota)	0.3% nanocellulose prepared by microbial hydrolysis (Mandel’s medium)	~0.2 IU/mL (Day 5)	Satyamurthy et al. [9]
*Trametes versicolor* (L) Loyd (CTB 863A)	Birch wood (5% malt extract, 2% agar) (22 °C, 70% RH)	0.21 U/mL (42 days)	Irbe et al. [48]
*Trametes versicolor* (L) Loyd (CTB 863A)	Aspen wood (5% malt extract, 2% agar) (22 °C, 70% RH)	0.30 U/mL (42 days)	Irbe et al. [48]
*Trametes versicolor* (L) Loyd (CTB 863A)	Alder wood (5% malt extract, 2% agar) (22 °C, 70% RH)	0.29 U/mL (42 days)	Irbe et al. [48]
*Trametes versicolor* CTB 863	Pine wood (MEA media, agar, colonized with ring) (22 °C, 70% RH)	0.25 ± 0.02 U/mL (10 days)	Elisashvili et al. [49]
*Trametes versicolor* CTB 863	Pine wood (WB media, agar, colonized with ring) (22 °C, 70% RH)	0.33 ± 0.01 U/mL (20 days)	Elisashvili et al. [49]
*Trametes versicolor* 159 (Basidiomycota)	TEMPO-oxidized-CNF(0.017 wt%) and CNC(0.2 wt%) (2% (*w/v*) ME, 25 °C, 80% RH) (standing cultures)	~ 20 ± 5 U/mL (25 days)	This study
*Trametes versicolor* 159 (Basidiomycota)	TEMPO-oxidized CNF(0.017 wt%) and CNC(0.2 wt%) (2% (*w/v*) ME, 120 rpm 25 °C, 80% RH)	~ 6 ± 2 U/mL (14 days)	This study
*Trametes pubescens* 220 (Basidiomycota)	TEMPO-oxidized CNF(0.017 wt%) and CNC(0.2 wt%) (2% (*w/v*) ME, 25 °C, 80% RH) (standing cultures)	~ 16 ± 3 U/mL (25 days)	This study
*Trametes pubescens* 220 (Basidiomycota)	TEMPO-oxidized CNF(0.017 wt%) and CNC(0.2 wt%) (2% (*w/v*) ME, 120 rpm, 25 °C, 80% RH)	~ 8 ± 4 U/mL (14 days)	This study
*Ganoderma adspersum* 003 (Basidiomycota)	TEMPO-oxidized CNF(0.017 wt%) and CNC(0.2 wt%) (2% (*w/v*) ME, 25 °C, 80% RH) (standing cultures)	~ 34 ± 2 U/mL (25 days)	This study
*Ganoderma adspersum* 003 (Basidiomycota)	TEMPO-oxidized CNF(0.017 wt%) and CNC(0.2 wt%) (2% (*w/v*) ME, 120 rpm, 25 °C, 80% RH)	~23 ± 2 U/mL (14 days)	This study
*Ganoderma lipsiense* 646 (Basidiomycota)	TEMPO-oxidized CNF(0.017 wt%) and CNC(0.2 wt%) (2% (*w/v*) ME, 25 °C, 80% RH) (standing cultures)	~ 42 ± 9 U/mL (25 days)	This study
*Ganoderma lipsiense* 646 (Basidiomycota)	TEMPO-oxidized CNF(0.017 wt%) and CNC(0.2 wt%) (2% (*w/v*) ME, 120 rpm, 25 °C, 80% RH)	~ 16 ± 4 U/mL (25 days)	This study
*Rigidoporus vitreus* 643 (Basidiomycota)	TEMPO-oxidized CNF(0.017 wt%) and CNC(0.2 wt%) (2% (*w/v*) ME, 25 °C, 80% RH) (standing cultures)	~ 21 ± 2 U/mL (25 days)	This study
*Rigidoporus vitreus* 643 (Basidiomycota)	TEMPO-oxidized CNF(0.017 wt%) and CNC(0.2 wt%) (2% (*w/v*) ME, 120 rpm, 25 °C, 80% RH)	~ 15 ± 5 U/mL (25 days)	This study

RH refers to relative humidity; ME refers to Malt Extract.

**Table 2:** Comparison of laccase activities of fungi grown in the presence of various lignocellulosic substrates.

Fungus	Growth Conditions	Laccase Activity	Study
*Trametes versicolor* IBB 897	Glucose medium, submerged fermentation containing mandarin peels (25 °C, 150 rpm)	3008 ± 325 U/L (10 days)	Elisashvili et al. [12]
*Trametes versicolor* IBB 897	Glucose medium, submerged fermentation containing tree leaves (25 °C, 150 rpm)	769 ± 84 U/L (10 days)	Ellisashvili et al. [12]
*Trametes versicolor* IBB 897	Glucose medium, submerged fermentation containing apple peels (25 °C, 150 rpm)	540 ± 59 U/L (10 days)	Elisashvili et al. [12]
*Trametes versicolor* IBB 897	Glucose medium, submerged fermentation containing banana peels (25 °C, 150 rpm)	1294 ± 149 U/L (10 days)	Elisashvili et al. [12]
*Trametes versicolor* (CBS100.29)	Glucose medium, containing barley bran (30 °C, 150 rpm)	639 U/L (37 days)	Lorenzo et al. [52]
*Trametes versicolor* (CBS100.29)	Glucose medium, containing grape stalks (30 °C, 150 rpm)	450 U/L (37 days)	Lorenzo et al. [52]
*Trametes versicolor* (CBS100.29)	Glucose medium, containing grape seeds (30 °C, 150 rpm)	250 U/L (37 days)	Lorenzo et al. [52]
*Trametes versicolor* 775	Basal synthetic medium containing CMC (180 rpm, RT)	131 ± 3.7 U/L (5 days) 136 ± 12.9 U/L (8 days)	Mikiashvili et al. [55]
*Trametes versicolor* 775	Basal synthetic medium containing maltose (180 rpm, RT)	178 ± 3.4 U/L (5 days) 95 ± 3.6 U/L (8 days)	Mikiashvili et al. [55]
*Trametes versicolor* 775	Basal synthetic medium containing Avicel (180 rpm, RT)	48 ± 2.7 U/L (5 days) 30 ± 2.5 U/L (8 days)	Mikiashvili et al. [55]
*Trametes versicolor* 775	Basal synthetic medium containing Cellobiose (180 rpm, RT)	663 ± 22.2 U/L (5 days) 742 ± 29.8 U/L (8 days)	Mikiashvili et al. [55]
*Trametes versicolor* 775	Basal synthetic medium containing mandarin peels (180 rpm, RT)	5243 ± 113 U/L (5 days) 3438 ± 80.9 U/L (8 days)	Mikiashvili et al. [55]
*Trametes versicolor* 145	Basal synthetic medium containing CMC (180 rpm, RT)	27 ± 2.7 U/L (5 days) 35 ± 2.8 U/L (8 days)	Mikiashvili et al. [55]
*Trametes versicolor* 145	Basal synthetic medium containing maltose (180 rpm, RT)	69 ± 10.3 U/L (5 days) 48 ± 3.5 U/L (8 days)	Mikiashvili et al. [55]
*Trametes versicolor* 145	Basal synthetic medium containing Avicel (180 rpm, RT)	15 ± 0.1 U/L (5 days) 11 ± 0.5 U/L (8 days)	Mikiashvili et al. [55]
*Trametes versicolor* 145	Basal synthetic medium containing Cellobiose (180 rpm, RT)	34 ± 4.1 U/L (5 days) 26 ± 3.4 U/L (8 days)	Mikiashvili et al. [55]
*Trametes versicolor* 145	Basal synthetic medium containing mandarin peels (180 rpm, RT)	428 ± 19.5 U/L (5 days) 79 ± 2.2 U/L (8 days)	Mikiashvili et al. [55]
*Trametes versicolor* (L) Loyd (CTB 863A)	Birch wood (5% malt extract, 2% agar) (22 °C, 70% RH)	0.01 U/mL (42 days)	Irbe et al. [48]
*Trametes versicolor* (L) Loyd (CTB 863A)	Aspen wood (5% malt extract, 2% agar) (22 °C, 70% RH)	0.01 U/mL (42 days)	Irbe et at. [48]
*Trametes versicolor* (L) Loyd (CTB 863A)	Alder wood (5% malt extract, 2% agar) (22 °C, 70% RH)	0 U/mL (42 days)	Irbe et al. [48]
*Trametes versicolor* (Empa strain 159)	Basal synthetic medium containing glucose and veratryl alcohol (25 °C, standing cultures)	122 ± 24 U/L (9 days)	Ihssen et al. [53]
*Trametes versicolor* (Empa strain 159)	Basal synthetic medium containing wood spruce dust (25 °C, standing cultures)	198 ± 44 U/L (9 days)	Ihssen et al. [53]
*Trametes versicolor* (Empa strain 159)	TEMPO-oxidized CNF(0.017 wt%) and CNC(0.2 wt%) (2% (*w/v*) ME, 25 °C, 80% RH) (standing cultures)	~351 ± 35 U/L (25 days)	This study
*Trametes versicolor* (Empa strain 159)	TEMPO-oxidized CNF(0.017 wt%) and CNC (0.2 wt%) (2% (*w/v*) ME, 25 °C, 80% RH, 120 rpm)	~225 ± 79 U/L (14 days)	This study
** Trametes pubescens (Empa strain 220)	Basal synthetic medium containing glucose and veratryl alcohol (25 °C, standing cultures)	282 ± 86 U/L (9 days)	Ihssen et al. [53]
** Trametes pubescens (Empa strain 220)	Basal synthetic medium containing wood spruce dust (25 °C, standing cultures)	53 ± 20 U/L (9 days)	Ihssen et al. [53]
*Trametes pubescens* (Empa strain 220)	TEMPO-oxidized CNF(0.017 wt%) and CNC(0.2 wt%) (2% (*w/v*) ME, 25 °C, 80% RH) (standing cultures)	~30 ± 16 U/L (25 days)	This study
*Trametes pubescens* (Empa strain 220)	TEMPO-oxidized-CNF(0.017 wt%) and CNC (0.2 wt%) (2% (*w/v*) ME, 25 °C, 80% RH, 120 rpm)	~110 ± 71 U/L (14 days)	This study
*Rigidoporus vitreus* (Empa strain 642)	Basal synthetic medium containing glucose and veratryl alcohol (25 °C, standing cultures)	2128 ± 252 U/L (9 days)	Ihssen et al. [53]
*Rigidoporus vitreus* (Empa strain 642)	Basal synthetic medium containing wood spruce dust (25 °C, standing cultures)	755 ± 148 U/L (9 days)	Ihssen et al. [53]
* Rigidoporus vitreus (Empa strain 643)	TEMPO-oxidized-CNF(0.017 wt%) and CNC(0.2 wt%) (2% (*w/v*) ME, 25 °C, 80% RH) (standing cultures)	~603 ± 75 U/L (25 days)	This study
* Rigidoporus vitreus (Empa strain 643)	TEMPO-oxidized-CNF(0.017 wt%) and CNC(0.2 wt%) (2% (*w/v*) ME, 25 °C, 80% RH, 120 rpm)	~238 ± 32 U/L (14 days)	This study
† Ganoderma lipsiense (Empa strain 646)	Basal synthetic medium containing glucose and veratryl alcohol (25 °C, standing cultures)	104 ± 18 U/L (9 days)	Ihssen et al. [53]
*† Ganoderma lipsiense* (Empa strain 646)	Basal synthetic medium containing wood spruce dust (25 °C, standing cultures)	18 ± 15 U/L (9 days)	Ihssen et al. [53]
*Ganoderma lipsiense* (Empa strain 646)	TEMPO-oxidized CNF(0.017 wt%) and CNC(0.2 wt%) (2% (*w/v*) ME, 25 °C, 80% RH) (standing cultures)	~4 ± 0 U/L (14 days)	This study
*Ganoderma lipsiense* (Empa strain 646)	TEMPO-oxidized CNF(0.017 wt%) and CNC(0.2 wt%) (2% (*w/v*) ME, 25 °C, 80% RH, 120 rpm)	~2 ± 1 U/L (11 days)	This study
*¥ Ganoderma adspersum* (Empa strain 003)	TEMPO-oxidized CNF(0.017 wt%) and CNC(0.2 wt%) (2% (*w/v*) ME, 25 °C, 80% RH) (standing cultures)	~95 ± 31 U/L (25 days)	This study
*¥ Ganoderma adspersum* (Empa strain 003)	TEMPO-oxidized CNF(0.017 wt%) and CNC(0.2 wt%) (2% (*w/v*) ME, 25 °C, 80% RH, 120 rpm)	~19 ± 14 U/L (25 days)	This study

RH refers to relative humidity; RT refers to room temperature; * *Rigidoporus vitreus* 643 is the same as 642 reported in Ihssen et al. 2011; ** *Trametes pubescens* 220 is mislabeled in Ihssen et al. 2011 as 568; ¥ *Ganoderma adspersum* 646 is the same as strain 647 reported in Ihssen et al. 2011; † *Ganoderma lipsiense* 646 is mislabeled in Ihnssen et al. 2011 as *Ganoderma adspersum* 647.

## Supplementary Materials

The following are available online at https://www.mdpi.com/2309-608X/7/3/222/s1, Figure S1: Growth rates of *T. versicolor* 159 in the presence of different percentages of malt extract (ME) and glucose (GL), Figure S2: Growth rates of *T. pubescens* 220 in the presence of different percentages of malt extract (ME) and glucose (GL), Figure S3: Growth rates of *G. adspersum* 003 in the presence of different percentages of malt extract (ME) and glucose (GL), Figure S4: Growth rates of *G. lipsiense* 646 in the presence of different percentages of malt extract (ME) and glucose (GL), Figure S5: Growth rates of *R. vitreus* 643 in the presence of different percentages of malt extract (ME) and glucose (GL), Table S1: Various genes for laccase production found in the genome of *T. versicolor* and *T. pubescens* based on the UniProt server (uniprot.org, accessed on 15 February 2021).
