# Supplementary material for: Enzyme Activities of Five White-Rot Fungi in the Presence of Nanocellulose
Source: J Fungi (Basel). 2021 Mar 18;7(3):222. doi: 10.3390/jof7030222 (PMC8003285; doi:10.3390/jof7030222)
Supplement: Supplementary file 1 [file jof-07-00222-s001.zip › Table_S1.docx]

**Table S1.** Various genes for laccase production found in the genome of *T. versicolor* and *T. pubescens* based on the UniProt server (uniprot.org).

| No. | UniProt KB identifier | Gene annotation | UniProt protein name | Fungus |
| --- | --- | --- | --- | --- |
| 1 | Q12718 | LAC2_TRAVE | Laccase-2 | *T. versicolor* |
| 2 | Q12719 | LAC4_TRAVE | Laccase-4 | *T. versicolor* |
| 3 | Q12717 | LAC5_TRAVE | Laccase-5 | *T. versicolor* |
| 4 | Q96UT7 | Q96UT7_TRAVE | Laccase B | *T. versicolor* |
| 5 | Q96UK8 | Q96UK8_TRAVE | Laccase 1 | *T. versicolor* |
| 6 | Q5IR80 | Q5IR80_TRAVE | Laccase 1 | *T. versicolor* |
| 7 | O94222 | O94222_TRAVE | Laccase | *T. versicolor* |
| 8 | O13456 | O13456_TRAVE | Laccase | *T. versicolor* |
| 9 | O13448 | O13448_TRAVE | Laccase | *T. versicolor* |
| 10 | A0A5Q2UQV7 | A0A5Q2UQV7_TRAVE | Laccase | *T. versicolor* |
| 11 | Q8TFM1 | Q8TFM1_TRAVE | Laccase III | *T. versicolor* |
| 12 | A0A144KZD6 | A0A144KZD6_TRAVE | Laccase | *T. versicolor* |
| 13 | A0A144KZJ7 | A0A144KZJ7_TRAVE | Laccase | *T. versicolor* |
| 14 | A0A144KZF3 | A0A144KZF3_TRAVE | Laccase | *T. versicolor* |
| 15 | A0A481SVG9 | A0A481SVG9_TRAVE | Laccase | *T. versicolor* |
| 16 | I6QS85 | 6QS85_TRAVE | Laccase | *T. versicolor* |
| 17 | Q50JG4 | Q50JG4_TRAVE | Laccase3 | *T. versicolor* |
| 18 | Q50JG3 | Q50JG3_TRAVE | Laccase4 | *T. versicolor* |
| 19 | Q50JG5 | Q50JG5_TRAVE | Laccase2 | *T. versicolor* |
| 20 | Q50JG6 | Q50JG6_TRAVE | Laccase1 | *T. versicolor* |
| 21 | G9M4T7 | G9M4T7_TRAVE | Laccase 5 | *T. versicolor* |
| 22 | Q6KB01 | Q6KB01_TRAVE | Laccase 4 | *T. versicolor* |
| 24 | B8Y3J5 | B8Y3J5_TRAVE | Laccase protein | *T. versicolor* |
| 26 | Q8TG94 | Q8TG94_TRAPU | Laccase 2 | *T. pubescens* |
| 27 | Q8TG93 | Q8TG93_TRAPU | Laccase 1A | *T. pubescens* |
| 28 | A0A1M2VSU5 | A0A1M2VSU5_TRAPU | Laccase-1 | *T. pubescens* |
| 29 | A0A1M2VJ73 | A0A1M2VJ73_TRAPU | Laccase-4 | *T. pubescens* |
| 30 | A0A1M2VJA1 | A0A1M2VJA1_TRAPU | Laccase-5 | *T. pubescens* |
| 31 | A0A1M2W0U1 | A0A1M2W0U1_TRAPU | Laccase-3 | *T. pubescens* |
| 32 | A0A1M2W307 | A0A1M2W307_TRAPU | Laccase | *T. pubescens* |
| 33 | A0A1M2VWR1 | A0A1M2VWR1_TRAPU | Laccase-2 | *T. pubescens* |
